# The Use of Contrast-enhanced Ultrasound in Pediatrics: A Case Series

**DOI:** 10.7759/cureus.6215

**Published:** 2019-11-21

**Authors:** Urban Čizmarević, Nina Hanžič, Matija Žerdin

**Affiliations:** 1 Radiology, University Medical Centre Maribor, Maribor, SVN

**Keywords:** contrast-enhanced ultrasound (ceus), pediatric radiology, kidney trauma, non-hodgkin lymphoma, kidney cysts, retropharyngeal abscess, necrotizing pancreatitis, vesicoureteral reflux

## Abstract

As the use of contrast-enhanced ultrasound (CEUS) is still relatively novel but quickly expanding, we would like to present a series of clinical cases where CEUS was used in diagnostics of pediatric patients. The presented cases include kidney trauma, non-Hodgkin’s lymphoma with secondary liver deposits, renal cortical cysts, retropharyngeal abscess, necrotizing pancreatitis, and vesicoureteral reflux. The wide range of the presented cases demonstrates CEUS' multipurpose use, which, together with its other useful attributes, especially its favorable safety profile, makes it an excellent diagnostic tool.

## Introduction

The use of contrast media with CT and MRI is widespread, with an estimated one-third to one-half of all investigations using it [1-3]. On the other hand, the use of contrast media with ultrasound (US) is still relatively new, without many official indications and thus mostly used "off-label." This is especially true for its use in pediatrics, but quickly changing as contrast-enhanced ultrasound (CEUS) as it continues to prove its worth, and the bureaucratic hurdles are slowly getting cleared. The contrast agents used with CEUS are microbubbles consisting of a small sphere of gas (e.g., sulfur hexafluoride), stabilized by a thin shell layer (usually a biocompatible lipid). The presence of bubbles in the vasculature changes the acoustic properties of tissues, increasing their echogenicity by 500-1000 times, resulting in a very strong reflection of the US waves [4]. The most commonly used agent in Europe is SonoVue, produced by Bracco International. The small size of the bubbles (1-7 µm; e.g., erythrocytes are 7-8 µm) makes sure that the bubbles stay intravascular and can circulate freely within the systemic vasculature [4]. The size also restricts the contrast from diffusing into the parenchyma and prevents glomerular filtration. Therefore, there is no contrast excretion in the urine; instead, the gas fraction is excreted with breathing, and the shell is metabolized in the liver [5]. This makes them safe for patients with renal insufficiency in which magnetic resonance (MR) and radiopaque CT agents are relatively contra-indicated [6]. An additional advantage is a high temporal resolution, which allows for good imaging of the contrast flow dynamics. Thus we can observe in real-time how quickly the targeted tissue fills with the contrast ("wash-in") and how long (from the point of full enhancement) it takes for the contrast to clear out ("wash-out"). These characteristics result in a high contrast image, which excellently shows the perfusion of the targeted tissue. We will try to demonstrate the value and our experiences with CEUS in the following pediatric examples. The videos are of the actual investigations of the presented cases.

## Case presentation

Case 1: Kidney trauma

**Figure 1:**
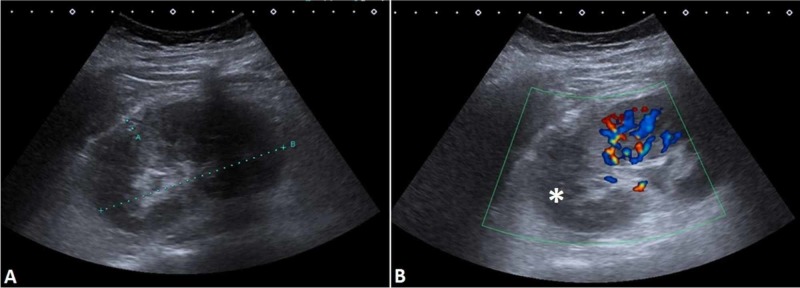
(A) B-mode US of the affected kidney. (B) Color Doppler demonstrated a lack of blood flow in the upper pole of the kidney (asterisk). US, ultrasound

An 11-year-old girl got injured during a fall with a bike. A day later, she started to experience abdominal pain and nausea. She has had a fever (38°C) and a sore throat for a few days before the injury. The inflammatory markers were increased [C-reactive protein (CRP) 133 mg/L]. The physical exam found tenderness in the costovertebral angle and the left upper quadrant of the abdomen. A therapy with co-amoxiclav was started. The B-mode US examination showed a hyperechogenic change in the left kidney located between the upper and middle thirds of the kidney (Figure 1A). A subcapsular hematoma was also described. Color Doppler showed blood flow only in the middle and lower thirds of the kidney (Figure 1B), pointing towards a laceration and infarction of the upper pole of the kidney.

A day after admission, the patient's inflammatory parameters began to rise (CRP 187 mg/L), and microscopic hematuria was detected. Chest radiograph and urine culture tests ruled out respiratory and urinary tract infections, and the focus of possible infection was not found. Co-amoxiclav was replaced with ceftriaxone. A control CEUS examination showed a devascularization of the upper third of the kidney parenchyma, which was confirmed by the MRI of the abdomen. Approximately 40% of the kidney parenchyma was affected, and the increase in CRP was presumed to be due to the ischemia/necrosis of the damaged part of the kidney. The patient was followed clinically and with US until discharge.

**Figure 2:**
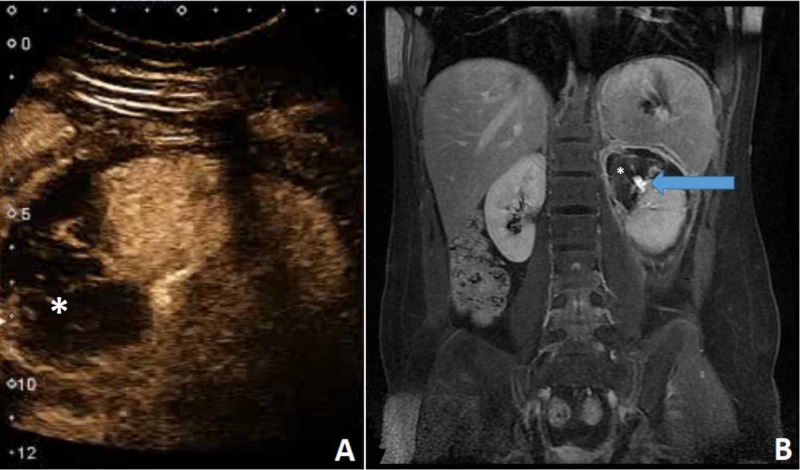
CEUS (A) showed good correlation of vascular defect (asterisk) compared to MRI (B). MRI additionally showed the urinary excretion (arrow). CEUS, contrast-enhanced ultrasound MRI, magnetic resonance imaging

In this case, CEUS showed the extent of laceration a lot more clearly than B-mode and Doppler US. The approximate area of the injury was detectable by color Doppler, but CEUS demonstrated the lesion better and helped to evaluate the degree of organ injury (Figure 2A). Renal parenchyma has very high blood flow, and thus, large quantities of contrast are visible quickly [7]. Due to the absence of the vasculature, the infarcted area appears hypoechogenic in all the phases and is clearly delineated from the perfused parts. When we compared it with MRI (Figure 2B), it showed a good correlation of the extent of the parenchymal injury, but due to the intravascular nature of US contrast, it did not show the minimal excretion of urine seen on the MRI.

It is not always possible to have an image of the kidneys as clear as in our case as the view can often be distorted by the ribs. This is especially true for the more superior positioned left kidney.

We agree with Miele et al., who think that CEUS in pediatric trauma could be used as first-line in mild blunt abdominal trauma, but not for major trauma or polytrauma where CT should be performed [8]. Additionally, if hematuria is present, CT should always be done, as due to the lack of glomerular filtration, CEUS is unable to detect a potential urinary tract injury (Video 1).

**Video 1:** CEUS - kidney trauma. CEUS, contrast-enhanced ultrasound

Case 2: Non-Hodgkin’s lymphoma and secondary liver deposits

**Figure 3:**
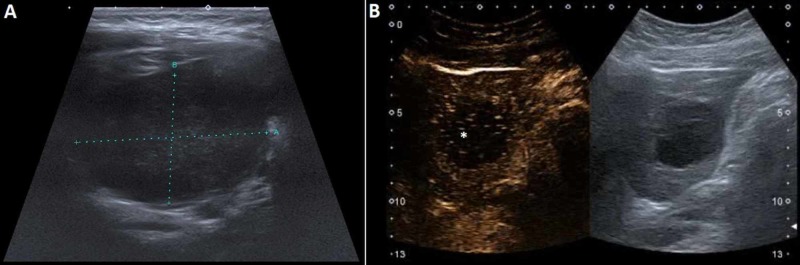
(A) US of the neck showed a 3 cm x 3 cm x 4 cm hypoechogenic change with a slightly heterogenic content. (B) CEUS confirmed the presence of microcirculation in the neck lesion (asterisk). US, ultrasound CEUS, contrast-enhanced ultrasound

A 13-year-old boy was admitted with a short history. Six days before the arrival, he noticed a painless, fast-growing mass on the right side of the neck. For the last two days, he was experiencing night-sweating, and he became febrile. He supposedly also lost some weight in the last week. On admission, he was in no distress and without any pain. Submandibularly a firm, immovable, painless mass, the size of 4 cm x 5 cm was palpable. The skin above the change showed no signs of inflammation. An enlarged lymph node was palpable in the axilla, measuring around 2 cm x 1 cm. There was no hepato- or splenomegaly. Blood count was within normal limits. Erythrocyte sedimentation rate was slightly increased. Biochemistry exams showed increased CRP, lactate dehydrogenase (LDH), ferritin, and urate. A chest radiograph did not show any masses or enlarged lymph nodes. The US of the neck showed a 3 cm x 3 cm x 4 cm hypoechogenic change with a slightly heterogenic content (Figure 3A). The use of Doppler did not convincingly show blood perfusion centrally, but it was evident at the periphery of the lesion. The differential diagnosis included a pathologic lymph node and a cystic lesion with echogenic content. The CEUS confirmed the presence of microcirculation in the neck lesion (Figure 3B), thus excluding a cystic lesion and pointing towards a pathological lymph node.

**Figure 4:**
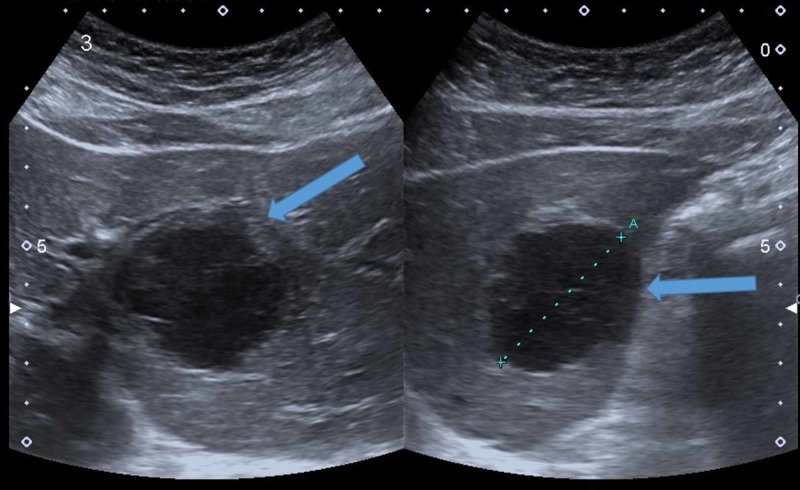
Abdominal US discovered two hypoechogenic changes in the liver (arrows). US, ultrasound

The abdominal US discovered two hypoechogenic changes in the liver (Figure 4). One measured 4.5 cm and the other up to 1.5 cm; with Doppler US, none of them showed central perfusion. Their content was not completely anechogenic. The larger one contained linear structures centrally -- possibly septae.

**Figure 5:**
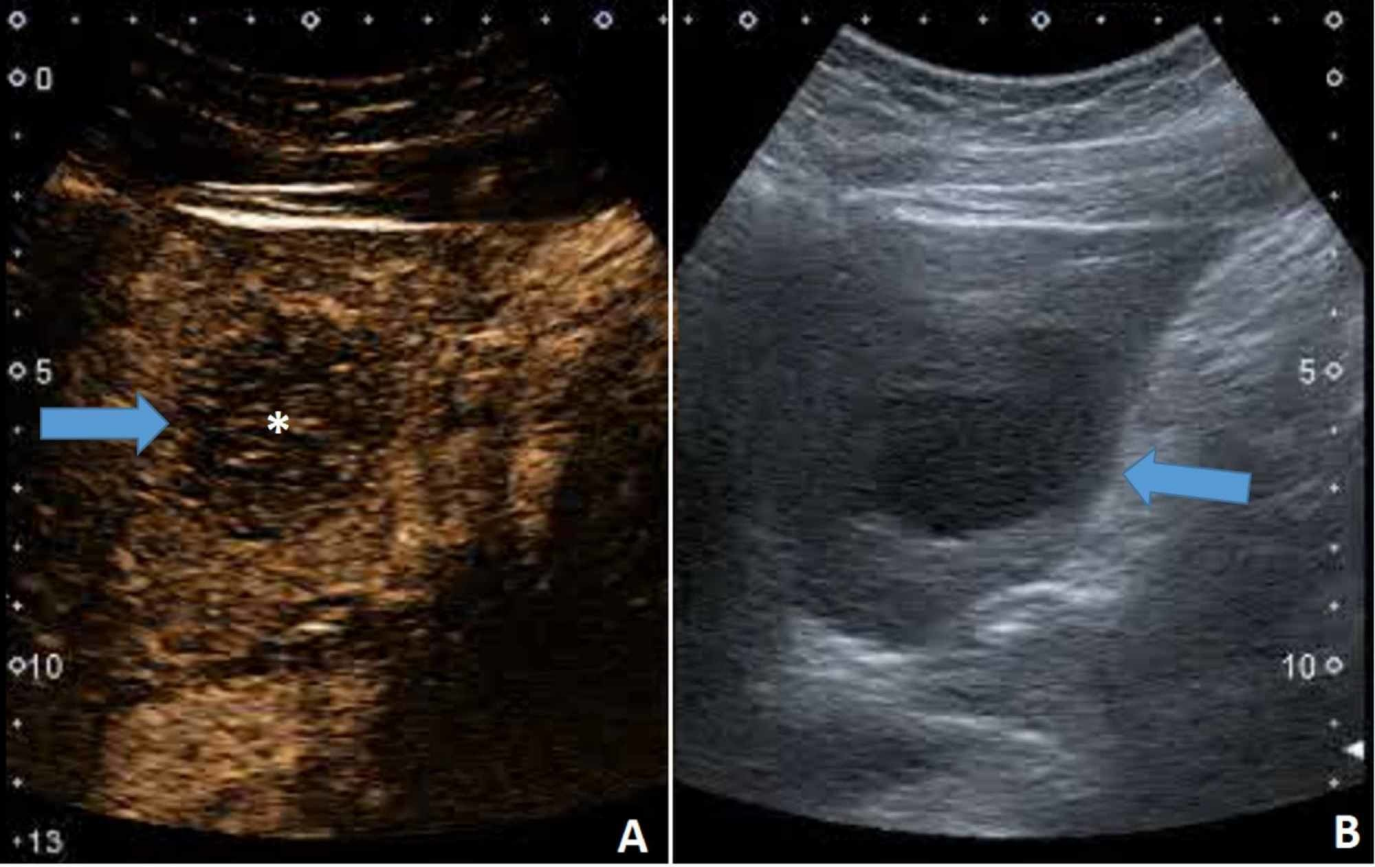
CEUS (A) demonstrating liver lesion's (arrow) microvasculature (asterisk) and fast washout. (B) B-mode US of the liver lesion (arrow). CEUS, contrast-enhanced ultrasound US, ultrasound

With the use of CEUS (Figure 5), a pathological filling pattern of the liver lesions was demonstrated with fast wash-in and fast wash-out, thus suspecting for a metastasis. The spleen was enlarged -- 13.5 cm x 6.5 cm, but structurally normal.

Fine-needle aspiration of the neck lesion was performed. A cytological examination with immunophenotypization was suspected for a non-Hodgkin’s lymphoma CD10+. Differential diagnosis included a Burkitt lymphoma or a diffuse large B-cell lymphoma. Later, the histological examination confirmed Burkitt lymphoma. The boy was treated according to the protocol.

In this case, CEUS excluded a cystic lesion of the neck, which was not possible using Doppler US alone. It also helped to evaluate the liver lesions as definitely pathological, most likely as metastases. Different lesions show different contrast filling patterns; however, in general, malignant lesions (because of their arterial neovasculature) fill with contrast already in the arterial phase and clear out faster than the surrounding parenchyma (Videos 2-3).

**Video 2:** CEUS - Non-Hodgkin's lymphoma. CEUS, contrast-enhanced ultrasound

**Video 3:** CEUS - secondary liver deposits. CEUS, contrast-enhanced ultrasound

Case 3: Cortical cysts and subcapsular hematoma of the kidneys

**Figure 6:**
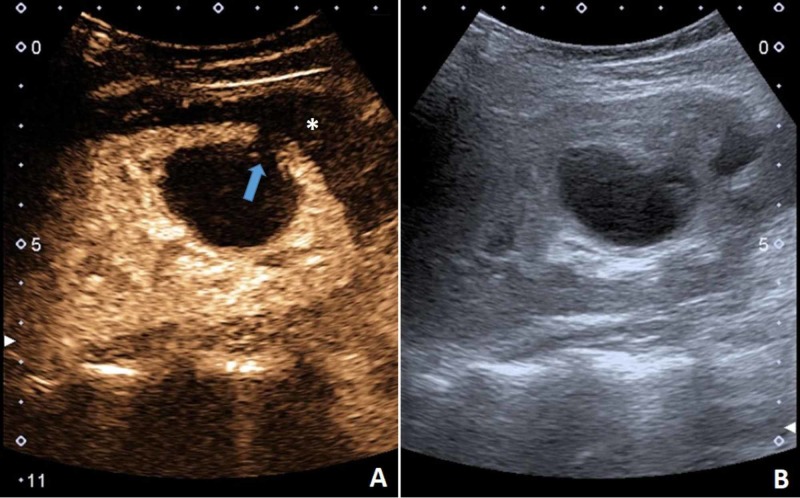
CEUS (A) demonstrating the larger cyst and its communication (arrow) with the subcapsular fluid collection (asterisk). (B) B-mode US. CEUS, contrast-enhanced ultrasound US, ultrasound

A 9-year-old boy had a bicycle accident and severely hit his left flank. Because of the abdominal pain and vomiting, internal organ injury was suspected. Abdominal US examination demonstrated a 3.5 cm x 3 cm hypoechogenic, possibly septated lesion in the lower pole of the kidney, as well as up to 15 mm thick layer of hypoechogenic material subcapsularly, probably a subcapsular hematoma. The following day US again showed the subcapsular hematoma, as well as three cortical cysts; two in the left kidney, measuring 4 cm and 8 mm, and one in the right kidney, measuring 12 mm. CEUS was performed for better delineation of the changes, and it showed homogenous parenchymal enhancement with a focal 8 mm large defect in the lower pole of parenchyma, through which the larger cyst communicated with the subcapsular fluid collection (most likely a mixture of hematoma and cystic fluid) (Figure 6). It also confirmed a subcapsular hematoma of the left kidney, located inferolaterally, measuring 7 mm laterally and up to 2 cm at the lower pole of the kidney.

The boy was hemodynamically stable, without micro- or macrohematuria, and was thus treated conservatively. Follow-up was done using US.

The CEUS helped us to delineate the parenchymal defect very well. It also showed a lack of active extravasation at the site of injury and the communication of the cyst with the subcapsular hematoma. Volume averaging used by CT can hide some thin septae, which may be seen with US and show enhancement with CEUS. As the evaluation of the malignant potential of the renal cysts is heavily influenced by the presence or absence of septae (Bosniak classification), CEUS can be very helpful in detecting early changes [9] (Video 4).

**Video 4:** CEUS - cortical cysts and subcapsular hematoma. CEUS, contrast-enhanced ultrasound

Case 4: Retropharyngeal abscess

**Figure 7:**
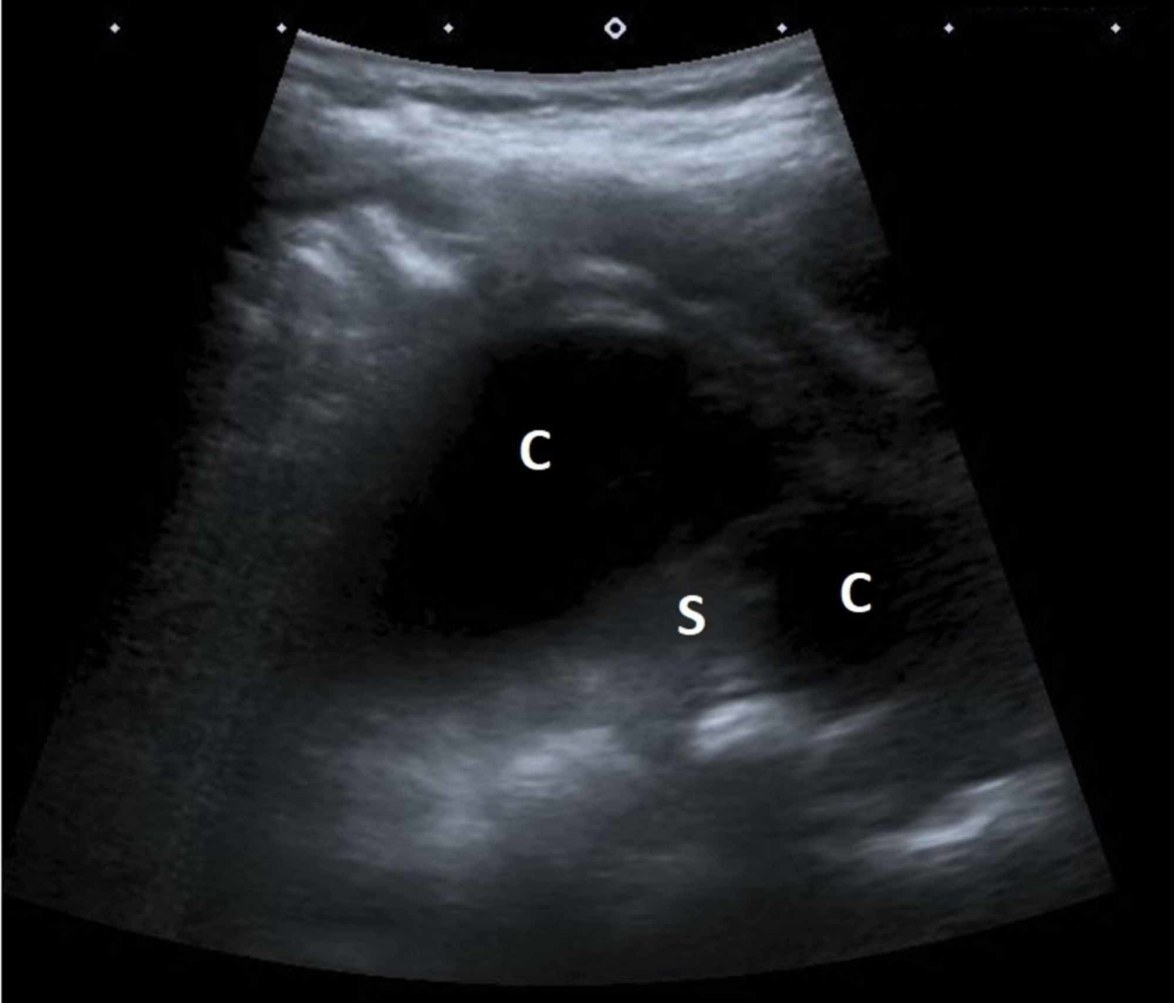
US examination of the neck showing a partly cystic (C) partly solid (S) change. US, ultrasound

An 8-month-old boy was treated for an abscessed lymph node of the neck, which was incised. Staphylococcus aureus was isolated, and he received the appropriate antibiotic therapy, after which he was asymptomatic and discharged home. His parents returned a month later after noticing the boy's tachypnea (40-50/min) and stridor. The physical exam revealed diffuse rales from the upper respiratory tract, without an obstructive component. Inflammatory parameters were elevated -- (CRP 20 mg/L, ESR 48 mm/h, leukocytes 28 x 109/L), but without neutrophilia or a left shift. He was afebrile. There was no palpable lymphadenopathy. Generally, the boy was lively and had a good appetite. Chest radiograph and abdominal US did not show signs of an infection. When searching for a possible focus of the infection an US examination of the neck revealed a partly cystic partly solid change, with the size of 4.5 cm x 3 cm x 5 cm to the left of the trachea (Figure 7).

**Figure 8:**
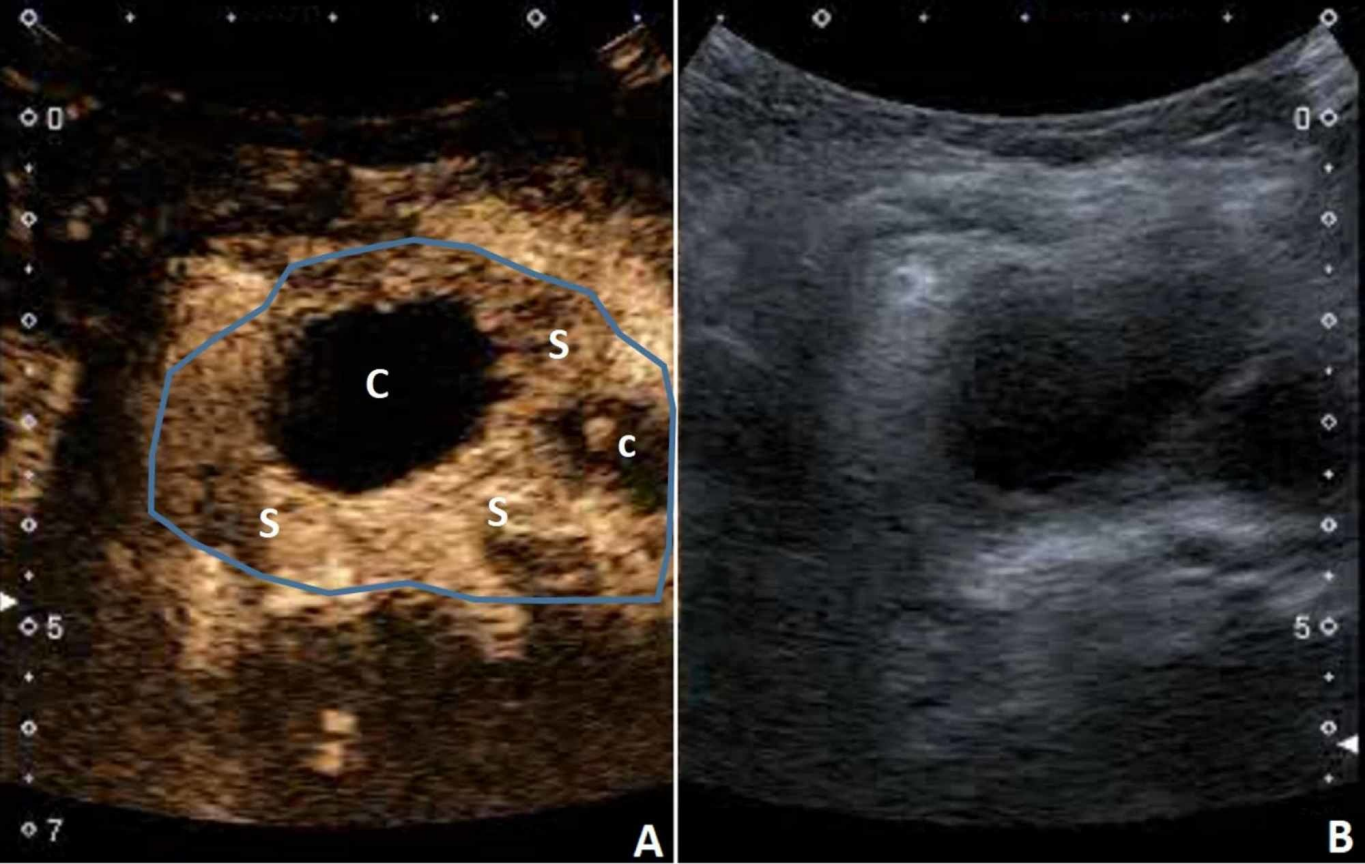
CEUS demonstrated peripheral enhancement of the mass (blue outline). Cystic parts (C) and solid parts (S) are clearly demarcated. (B) B-mode US of the neck mass. CEUS, contrast-enhanced ultrasound US, ultrasound

After contrast application, the finding enhanced peripherally -- a 1 cm thick band of tissue became apparent (Figure 8).

**Figure 9:**
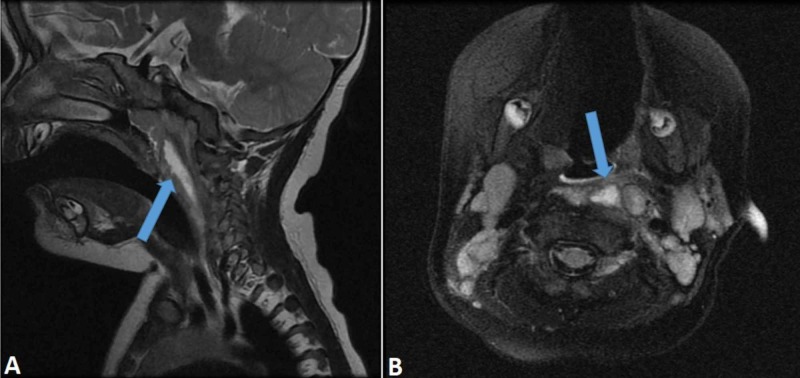
MRI of the neck confirming the presence of an abscess (arrow). (A) Sagittal view. (B) Axial view. MRI, magnetic resonance imaging

An MRI of the neck was performed and it confirmed a retropharyngeal abscess, which was spreading downwards along the prevertebral muscle (Figure 9).

Due to poor access to the abscess for an incision (small child with a very short neck), the ENT physicians decided for a conservative approach with a prolonged seven-week antibiotic treatment. The therapy led to a complete regression of the abscess and resolution of the breathing problems. The changes regressed quickly and were no longer visible with US; thus, MRI was used for the subsequent following. Due to two severe infections in such a short period, the immunological tests were done but were negative.

In this case, the visualization of the neck was hindered because of the patient's constitution and short neck. Therefore, we were only able to obtain axial images of the neck region. Nevertheless, the US was useful in detecting the clinically unexpected lesion of the neck. CEUS helped to narrow down the diagnosis, but the final diagnosis was made by MRI, which then had to be used for follow-up due to the lesion becoming too small for visualization on the US (Video 5).

**Video 5:** CEUS - retropharyngeal abscess. CEUS, contrast-enhanced ultrasound

Case 5: Necrotizing pancreatitis

**Figure 10:**
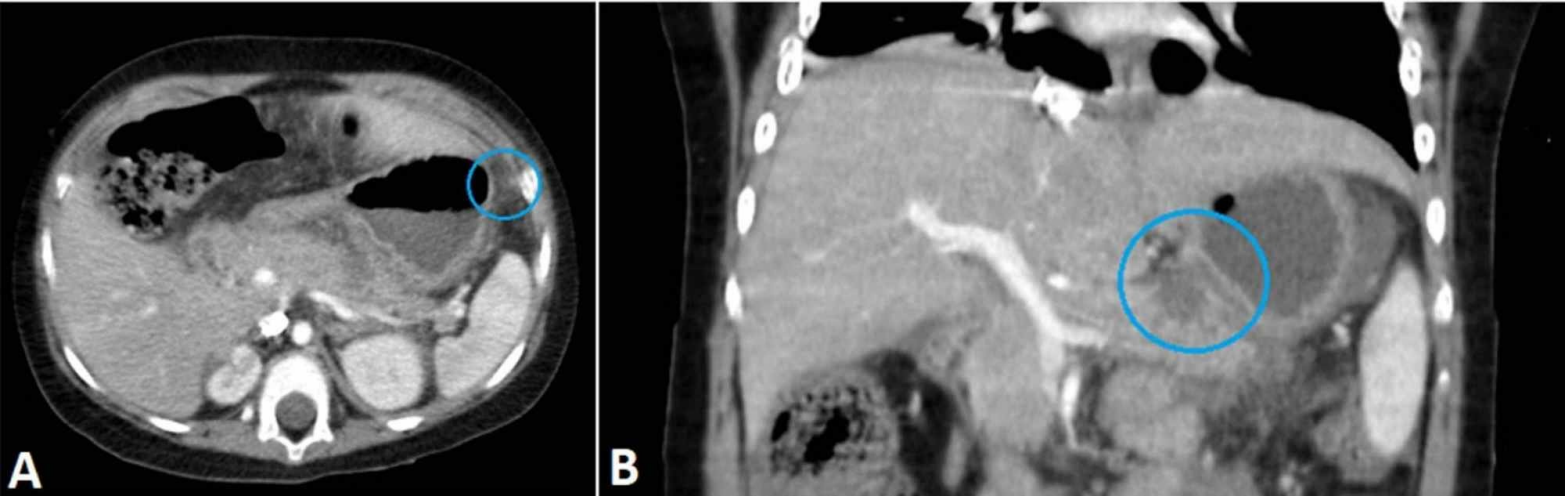
A CT examination suggestive of necrotizing pancreatitis. (A) Axial view. (B) Coronal view. CT, computed tomography

A 3-year-old girl with many comorbidities (including Miller-Dieker syndrome and West syndrome) was admitted to the department because of problems with the passage of food through a percutaneous endoscopic gastrostomy (PEG) tube. The parents reported that any food given via PEG tube remained in the stomach until the next feeding attempt. She was also in pain. The US examination detected a paralytic ileus and free fluid in the abdomen. The inflammatory parameters and serum amylase and lipase were significantly increased (CRP 353 mg/L, leukocytes 22.8 x 109/L , amylase 2.4 ukat/L, lipase 24.7 ukat/L). A CT scan showed an edematous pancreas, with an absence of focal opacification in the pancreatic tail, as well as inflamed peripancreatic fat, reactive mesenteric lymph nodes, and thickening of the adjacent stomach wall (Figure 10). There were neither signs of pancreatic duct obstruction, nor were there any peripancreatic fluid collections. The findings were consistent with necrotizing pancreatitis.

**Figure 11:**
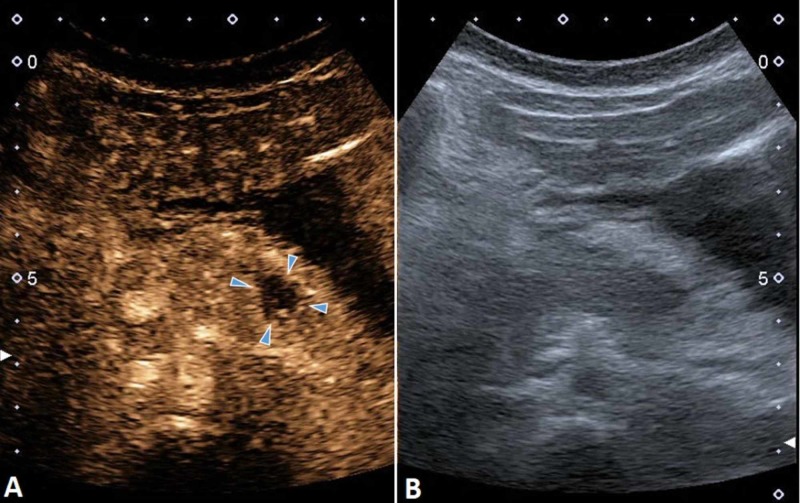
CEUS (A) demonstrating a 14 mm perfusion defect in the tail of the pancreas (arrowheads), which was not clearly seen on the B-mode US (B). CEUS, contrast-enhanced ultrasound US, ultrasound

The patient was put on nil per os (NPO) and was fed parenterally. Antibiotic therapy was started. She was then followed with US, which showed a relatively clear image of the pancreas, but the injured area was poorly contrasted from the healthy parenchyma. With the use of CEUS, we managed to show a 14 mm perfusion defect in the tail of the pancreas (Figure 11), so we continued the follow-up using US and CEUS.

After the antibiotic therapy, the girl improved, but two days after therapy completion, the inflammatory parameters increased again. This time, the US showed a peripancreatic fluid collection, but CEUS showed no additional findings in the pancreatic parenchyma. The antibiotic treatment was repeated, and this time she recovered uneventfully. The peripancreatic fluid collection disappeared spontaneously over a two-week period. A few months after discharge, the patient had an additional subclinical flare-up of amylase and lipase, which normalized after being kept on NPO again. This time, magnetic resonance cholangiopancreatography (MRCP) was done but it showed no abnormalities in pancreatic duct, common bile duct, or gallbladder.

The US was able to replace CT in this patient as the follow-up method because it was possible to visualize the pancreas very well in every examination. This was helped by the patient having a PEG tube, which allowed us to fill the stomach with fluid and remove any excess air. Usually, due to its retroperitoneal position, visualization of the pancreas can be very difficult. CEUS was useful in delineating the necrotic area of pancreatic parenchyma and was thus also useful in the follow-up of the patient (Video 6).

**Video 6:** CEUS - necrotizing pancreatitis. CEUS, contrast-enhanced ultrasound

Case 6: Vesicoureteral reflux

A pre-natal US examination of a baby girl showed hydronephrosis, megaureter, and expanded pyelo-ureteral connection of the right kidney. Postnatally it was clear that there was a duplicated collecting system with two ureters of the right kidney, dilatation of the upper pole, and dilatation of the second ureter, which drained into the bladder with ureterocele. Scintigraphy showed isotope buildup in the upper third of the kidney. Kidney function tests were within normal values. Prophylaxis with cefuroxime (1.25 mL in the evenings) against potential urinary tract infections (UTIs) was started. Contrast-enhanced voiding urosonography (ceVUS) showed evidence of fourth-degree vesicoureteral reflux (VUR) into the expanded ureter and the collecting system of the lower pole of the right kidney. Deflux surgery was performed (application of Vantris) and the ureterocele was incised, but not completely removed, as the surgeon felt it was too close to urethra and that complete removal might compromise the girl's continence. During the surgery, it was seen that the duplicated ureter drained either into the neck of the urinary bladder or into the proximal part of the urethra (where the ureterocele was). At the postsurgical examination, ceVUS showed third-degree reflux into the collecting system of the lower pole of the kidney. There was no reflux into the upper pole of the kidney. As the girl had a known duplication of the ureters on the right side, we performed cyclic ceVUS with two additional cycles and managed to show reflux also on the left side as well as duplication of at least part of the left ureter.

**Figure 12:**
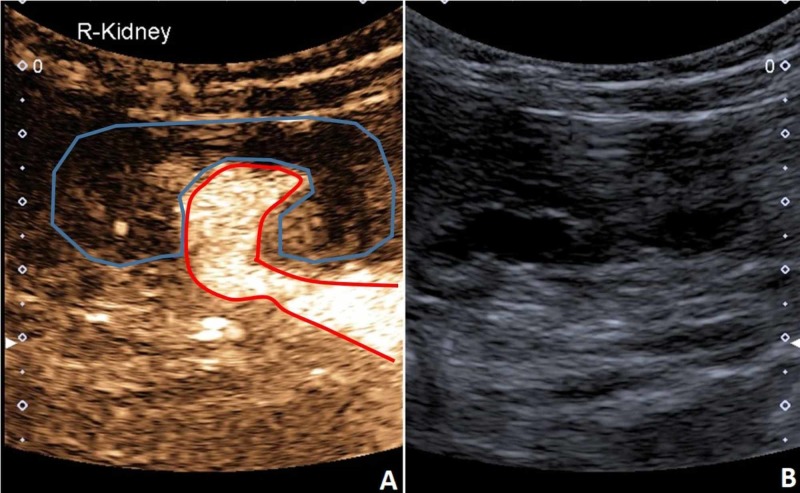
CEUS (A) demonstrating retrograde filling of the lower part of the duplicated kidney collecting system (red outline). Rough margins of the kidney (blue outline). (B) B-mode US of the kidney. CEUS, contrast-enhanced ultrasound US, ultrasound

The VUR is so far the only indication where US contrast is officially approved in the pediatric population in Europe, and its use is not off-label. A urinary catheter is inserted, and a contrast is injected. The kidneys, ureters, and bladder are then scanned sequentially before, during, and after voiding, and in case of VUR, a retrograde filling of the ureters and pyelon with microbubbles is observed. With a sensitivity and specificity of around 90%, ceVUS has been shown to be as efficient as voiding cystourethrography (VCUG) for the diagnosis of VUR. However, since with ceVUS, we avoid radiation exposure, it is seen as a preferable option when its use is possible. If the child is noncooperative, the intravesical pressure may rise and the contrast agent is destroyed quickly. Some children may also have problems with the postponed voiding phase. In these cases, VCUG may still have to be used [10-12] (Figure 12).

## Discussion

These are some of our more interesting examples in which CEUS played an important role in the patient diagnosis or follow-up. However, for all the advantages of CEUS, it also has several limitations. With US, the visualization of deep regions is often problematic, and the view is narrowly focused on a particular body part, unlike in other imaging methods, where we can acquire an overview of the entire regions. If we are unable to see a structure on B-mode US, then adding a contrast agent will not improve its visualization. CEUS should always be used as an addition to B-mode US and not as its replacement. Furthermore, the need for proficiency in US use is even more pronounced with CEUS because the contrast clears quickly and the examining window is short (up to 2 min for B-mode and 3-8 min for Doppler [13]). The off-label use necessitates a written consent from the parents, but that is usually not difficult to obtain once the procedure is explained.

The CEUS' main advantages are the avoidance of unnecessary radiation, anesthesia or restriction, as well as real-time demonstration of vascularization of pathological processes, and higher sensitivity in detecting vascularization [compared to contrast-enhanced CT (CECT) and contrast-enhanced magnetic resonance (CEMR)]. Additionally, the contrast agents used are proven to be at least as safe as the gadolinium-based contrasts and significantly safer than radiopaque contrasts [14]. Due to the strong signal of the US contrast agents, the doses are significantly smaller (few micrograms) than those used with CT and MR (few milligrams) [15].

## Conclusions

Owing to the lack of radiation exposure, CEUS is extremely valuable for patient follow-up, as it allows us to perform frequent check-ups. In trauma patients, it cannot be used as a standalone diagnostic method for parenchymal injuries, as it is usually not sensitive enough (because of US limitations in general) and can miss important details. However, it can be very useful for triage and guiding us in the right direction at the beginning of the investigations. All in all, though not without shortcomings, CEUS is an excellent tool in many cases, and its use will undoubtedly continue to grow.
